# A method for determining the recycling value of unprocessed municipal solid waste in one cubic meter waste composition analysis technique

**DOI:** 10.1016/j.mex.2024.102626

**Published:** 2024-03-09

**Authors:** N. Arosha Hemali, A.A.P. De Alwis, M. Wijesundara, L.G.L.M. Edirisinghe

**Affiliations:** a Department of Chemical and Process Engineering, University of Moratuwa, Moratuwa 10400, Sri Lanka; b Faculty of Post Graduate Studies, University of Moratuwa, Moratuwa 10400, Sri Lanka; c National Innovation Agency, Battaramulla 10120, Sri Lanka; d ANVARTA Insights Consulting Services Solutions, Sri Lanka

**Keywords:** Circular economy, Resource circulation, Waste symbiosis, A method for determining the recycling value of unprocessed Municipal Solid Waste in One Cubic meter Waste Composition analysis technique

## Abstract

The transition from conventional landfill-centric waste management to resource-centric methodologies necessitates an enhanced comprehension of municipal solid waste (MSW) composition and its inherent value. Existing methodologies documented in the literature exhibit a lack of standardization, impending the formulation of a systematic engineering approach for MSW characterization and valuation. This study introduces a methodology specifically tailored to discern the composition of waste origination from urban households and evaluate its recyclability within the confines of a circular economy framework, Employing a volume-based measurement approach, aims to estimate the recycling value of waste materials. The study's outcomes contribute significantly to quantifying the potential recycling value that accrues to society. Furthermore, the validation of the proposed protocol elucidates the dynamic nature of recyclable value as it traverses the intricate pathways of the waste supply chain. This insight facilitates the formulation of commercial models grounded in circular economy principles for the effective management of household solid waste. Empirical findings reveal that the total recycling value fluctuates within the range of USD 3.39 and USD 5.76 per cubic meter of waste volume, contingent upon the specific waste composition at the experiment site. Additionally, the proposed methodology uncovers the nuanced variability in MSW composition and recycling value across diverse household collection patterns, identifying mixed plastic, paper, cardboard, mixed MSW, and clothing as primary constituents. The application of this methodology extends beyond mere quantification, providing a foundational framework for simulating the latent recycling value embedded within MSW samples. This, in turn, offers invaluable support to strategy developers, policymakers, and entrepreneurial ventures engaged in the sustainable management of household solid waste. In essence, this study establishes the groundwork for a comprehensive understanding of MSW composition and its recyclability, facilitating informed decision-making in the pursuit of a circular economy.

• Novel methodology based on one cubic meter (1m3) composition analysis of Municipal Solid Waste (MSW).
• A new method to evaluate the recycling value of Municipal Solid Waste.
• A basis for business model development for the waste-to-resource conversion model.

## Graphical abstract

**Figure:**
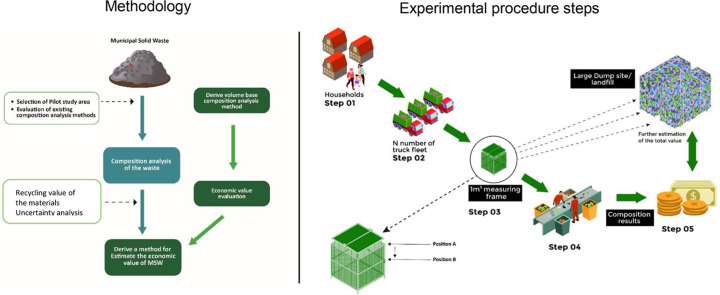

Specifications Table

**Table:** 

Subject area:	Environmental Science
More specific subject area:	Waste to value identification
Name of your method:	A method for determining the recycling value of unprocessed Municipal Solid Waste in One Cubic meter Waste Composition analysis technique
Name and reference of original method:	Not applicable
Resource availability:	Not applicable
Trial registration:	Not applicable

## Introduction

A large amount of municipal solid waste is deposited into landfills without considering its potential value to society, resulting in the squandering of valuable resources. Accurately assessing resource recovery from municipal solid waste necessitates reliable information on waste generation and composition. According to [1], over 50% of global waste ends up in open dumps (33%) and landfills (23.11%) without regard for potential market value. The escalating trend of household waste contributing to open dumpsites poses a significant challenge to sustainable resource consumption practices.

In 2021, the EU treated 234.0 million MTs of MSW, with material recycling constituting 30.1% and landfill disposal comprising 23.3% [2]. It was reported that global municipal solid waste generation in 2018 totaled 2.02 billion metric tons and is projected to increase by 70% to 3.4 billion metric tons by 2050 [3]. As per the 2022 Global Waste Index (GWI), the USA has emerged as the most waste-generating country, producing 811 kg per capita annually. Conversely, Turkey has been identified as the least environmentally friendly waste management country. Switzerland stands out as the sole nation not sending any waste to landfills [4].

The significance of understanding MSW composition lies in the necessity to assess material and energy recovery potentials. Comprehensive waste composition data is crucial for decision-making on waste utilization, developing efficient waste management systems, planning information, and advocacy campaigns. However, the absence of a reliable information-gathering procedure hampers results in comparisons between studies, making reliability and comparisons challenging. Waste composition studies with resource valuation models are pivotal in enhancing the functionality of waste management systems and reimagining resource circulation, constituting a prerequisite for any waste management application.

### Background and literature review

According to the literature review of methods for household waste composition studies, twenty such methods are listed and commented on in Table 1. There is no adopted, working international standard. The majority of the methods rely on the sample's mass, while others depend on the number of households or the flow of waste material [5]. Interestingly, only one study utilizes sample volume as a basis. In 2021, the waste composition analysis for Greece emphasized the use of the volume-measured approach, following the 2004 European Commission Solid Waste Management Tool [6]. Concurrently, the Minnesota Pollution Control Agency conducted a study, specifically focusing on volume-to-weight conversions of waste compositions. This study provided detailed information on the densities of solid waste streams and various sampling methodologies. Further ISO (International Organization for Standardization) 14,001 has yet to develop a specific solid waste composition analysis method. Furthermore, it is noticed that the ASTM composition analysis method (2016) [7] and the European Commission SWA tool (2004) [6] are the methods widely used over the last three decades. However, neither evaluation of the recycling value of the waste samples nor its substantiation has been supported.

**Table 1:** Summary of MSW composition test methods based on the basis for sampling as per [5].

Sampling basis	Method name
Mass	ASTM (2016), RVF (2005a), RVF (2005b), SAEFL (2004), CIWMB (1999), ADEME (1998), Rugg (1997), Reinhart and McCauley Bell (1996), Scott (1995)
Number of households	Burnley et al., (2007), Petersen (2004), Mbande (2003), Ohlsson (1998), Nordtest (1995), Cornelissen and Otte (1995), Gustafson and Johansson (1981)
The volume of waste bins	European Commission (2004)
Material flow method	Franklin and Associates (1999), Gay et al. (1993)

The study seeks to fill existing gaps in practices by introducing a novel approach for analyzing MSW based on volume measurements. That enables the determination of waste composition within a cubic meter volume and the quantification of its recycling value. Crucially, this approach facilitates the evaluation of MSW composition without disrupting the current waste state. It has identified previously unexplored opportunities for resource circularity and furnished valuable evidence on the recycling potential embedded within the waste.

The study was conducted at the MSW collection site (6°81′50.65″N 79°90′19.7″ E) Karadiyana in the Western province of Sri Lanka and studied from an urban waste generation perspective in a developing country.

## Experimental procedure

### Proposed method

**Fig. 1:**
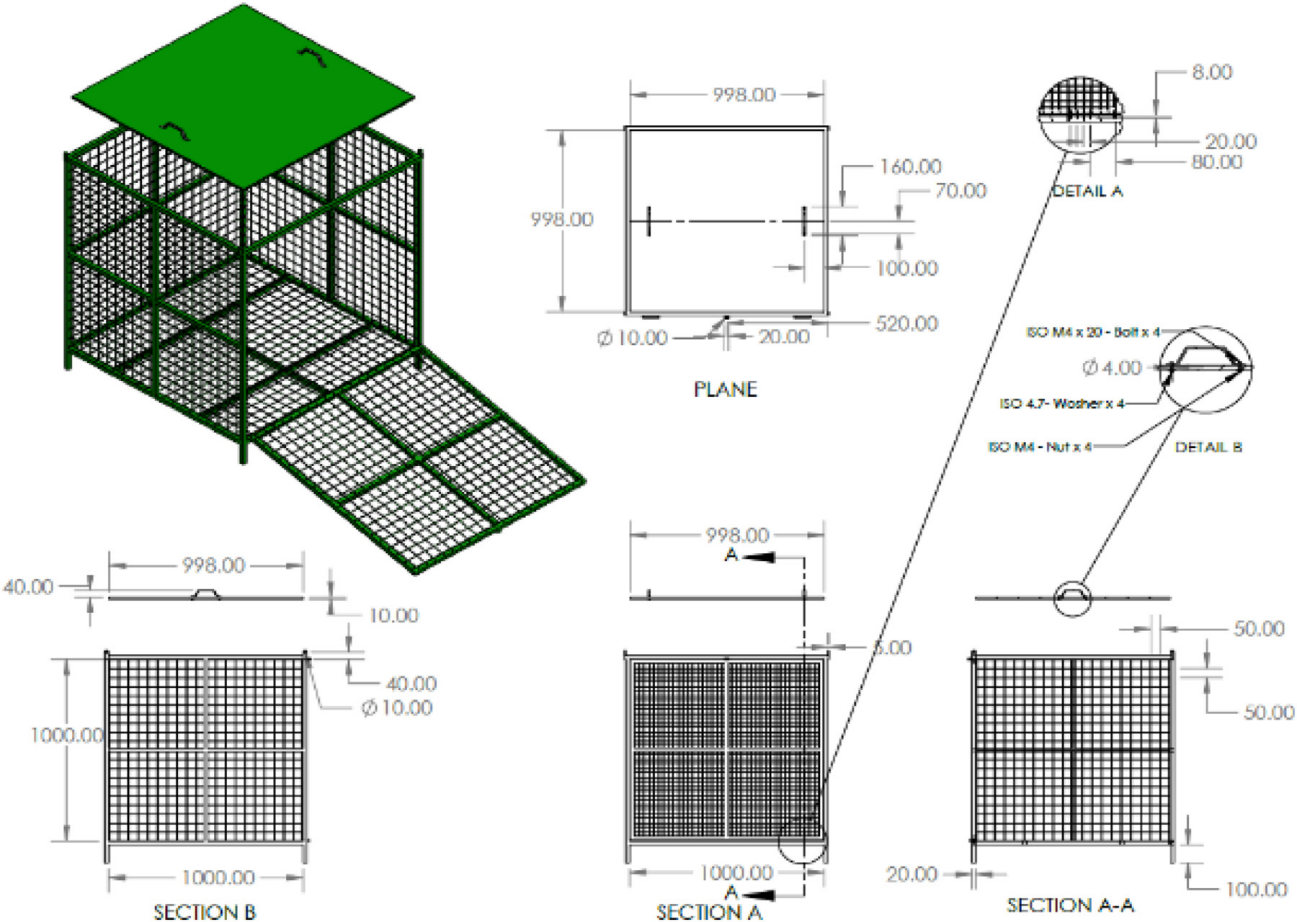
Design of the 1m3 vol waste measuring frame (metal cube).

The Biocube concept of biodiversity evaluation was supported for deriving the methodology. A biocube is an informative and manageable way of exploring the biodiversity in the world around by focusing on a cubic foot of space [8]. The same was experimented to identify the different waste characteristics in a specific volume. Therefore, the Biocube assessment of biodiversity characterization by [9] and [8] has supported the development of one cubic meter MSW composition analysis. The study used a customized 1m^3^ metal cube to illustrate the concept as shown in Fig. 1.

The volume size was chosen to directly link it with the density of the sample in the experiment. This will allow more accurate and representative MSW sample analysis. The key objective of the 1m^3^ method was to improve the precision and comparison of MSW composition. Using a uniform volume, the method intends to offer a more reliable and consistent basis for waste analysis in different municipal solid waste collection locations and valuing recycling potential.

### Scope

**Fig. 2:**
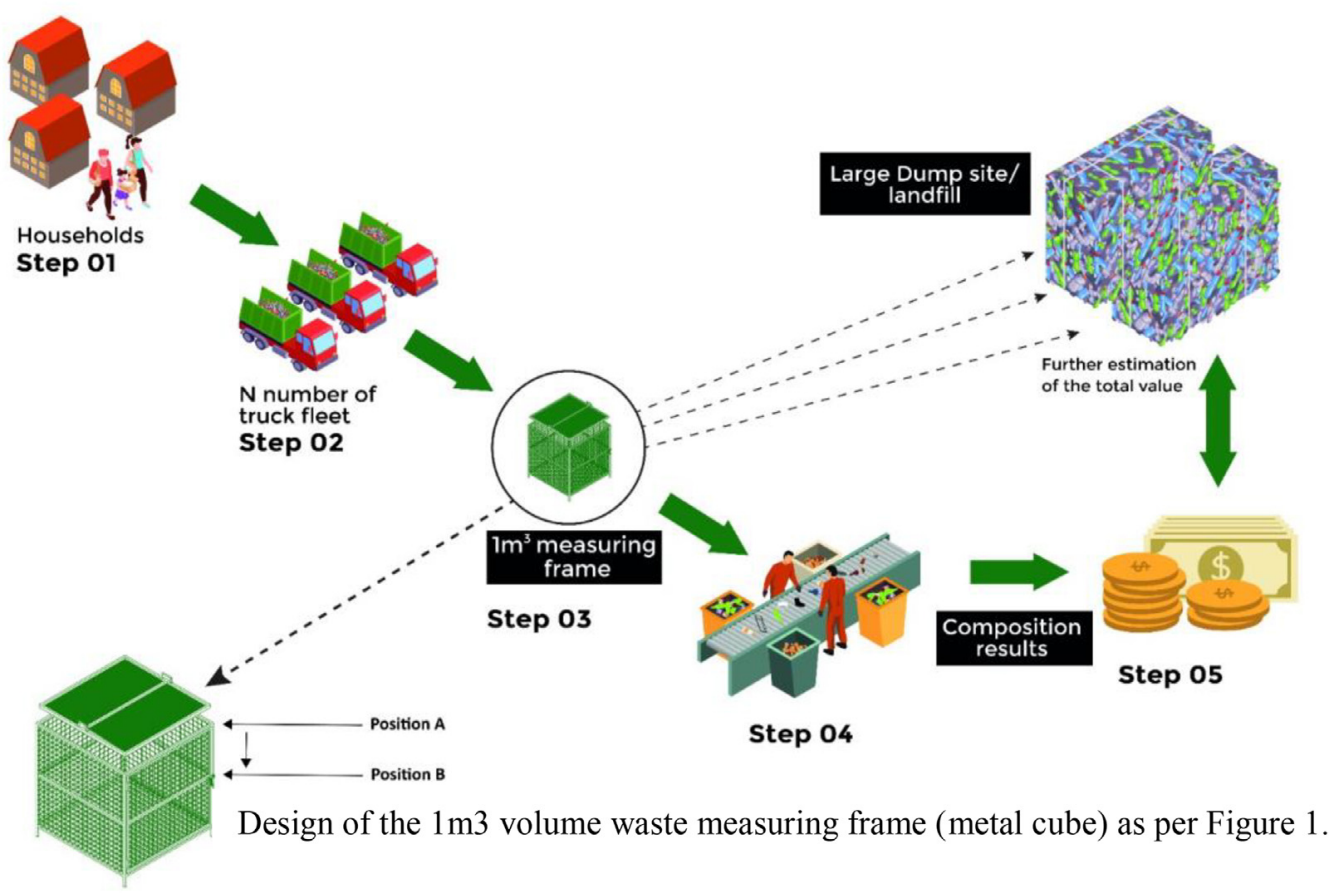
Sampling and composition analysis process in one cubic meter volume and extrapolation to the waste dump.

The proposed method includes procedures for collecting a representative sample of unprocessed waste, manual sorting of waste into individual waste components as per the steps stated in Fig. 2. The protocol proposes for measuring the composition of unprocessed MSW using manual sorting (step 4 of Fig. 2) and determination of the recycling value of the waste materials. It applies to the determination of the mean recycling value of the composition of MSW based on the collection sample in a standard volume of one cubic meter. The number of samples to be evaluated according to the statistical guidelines. Further data reduction and reporting results according to ASTM (2016) [6].

The proposed method can be applied to open dumps, waste-filled truck fleets, landfills, waste processing and conversion facilities, and transfer stations. The main sample is confined to a cubic-meter volume, and the composition is analyzed on a weight basis in units of kilograms because it is directly linked to density. Furthermore, the user of this method should establish appropriate safety and health practices and determine the applicability of regulatory limitations before use ASTM (2016) [6]. Adherence to these procedures ensures the consistency and accuracy of the waste composition results.

### Sample selection & preparation

The random sampling technique was used for field sampling preparation. Random sampling can be utilized as the basis for sampling as highlighted by [10]. However, the literature does not provide precise guidance on the number of samples required [11]. According to [12], sixty samples can be taken as the basis for waste characterization,

### Sample measuring

The experiment was carried out for fresh nonbiodegradable waste entering the municipal waste site from the waste trucks comprising household collected material, as shown in Fig. 2. In Step 1, mixed non-biodegradable waste is collected from bins at households. It is gathered by collection trucks and transported to the site at Karadiyana as part of step 2. Random samples from tucks are collected to one cubic meter metal frame in step 3. All the mixed waste then gets further mixed inside the frame.

The sample volume size in step 3 is selected because one cubic meter can represent the sample density in the experiment. Once the cube was filled with waste up to the maximum level of the cube (position A) of Fig. 2 (Design of the 1m3 vol waste measuring frame), it was closed using the lid fabricated from a metal plate 10 mm thick and weight of 7.8 kg. Then, as a result of the weight of the lid, the waste material is compressed into the cube, and the lid is placed in a new position (position B). Once again, the waste material is placed inside the cubic frame and secured with the lid positioned at position A. Subsequently, manually separate the materials into distinct categories and analyze the composition of the waste material contained within a 1 m³ volume in Step 4 and then converted to a recyclable value as in step 5. Waste categories can be taken as specified in ASTM (2016) [6] Table 2 or depending on the requirement. However, to derive recyclable value, categorization as much as possible would be advantageous.

**Table 2:** Description of the waste categories as per ASTM (2016) [6].

Category	Description
Mixed Paper	Office paper, computer paper, magazines, glossy paper, waxed paper, and other paper not fitting the categories of newsprint and corrugated
Newsprint	Newspaper
Corrugated	Corrugated medium, corrugated boxes or cartons, and brown (kraft) paper (that is, corrugated) bags
Plastic	All plastics
Yard waste	Branches, twigs, leaves, grass, and other plant material
Food waste	All food waste except bones
Wood	Lumber, wood products, pallets, and furniture
Other organics/ Combustibles	Textiles, rubber, leather, and other primarily burnable materials not included in the above component categories
Ferrous	Iron, steel, tin cans, and bi-metal cans
Aluminum	Aluminum, aluminum cans, and aluminum foil
Glass	All Glass
Other inorganics/non-combustibles	Rock, sand, dirt, ceramics, plaster, non-ferrous nonaluminum metals (copper, brass, etc.), and bones

The weight fraction of each component in the sorting sample was calculated from the weights of the components as per equation 2. The mean waste composition was calculated using the results of the composition of each of the sorting samples.

### Waste categories

Multiple categories were used to classify waste: paper and cardboard, plastic, wood, yard waste, Food waste, Other organic combustibles, ferrous, aluminum, glass, and other inorganics non-combustibles. The present study excludes biodegradable.

### Composition of materials

The composition of the waste material is implied in Equation 2 below.

(1) $$\text{Total}\;\text{Weight}\;\text{of}\;1\;\text{cubic}\;\text{meter}\left(W_{\text{total}\;}\right)\;=\text{Weight}\;\text{of}\;\left(W1+W2+W3+--------\text{Wn}\right)$$

Wi= Component i (i can be 1 to n number of components according to Table (2)

The weight is taken in kilograms (kg) form.

(2) $$\text{The}\;\text{composition}\;\text{of}\;\text{each}\;\text{component}\;\text{would}\;\text{be}=\;\left(\left(\text{Weight}\;\text{of}\;\text{Wi}\right)/\text{Weight}\;\text{of}\left(W_{\text{total}}\right)\right)*100\%$$

### Recycling value of waste

This study presents an alternative approach for assessing the recycling value of mixed waste with a volume of 1m^3^. One of the main objectives of this method is to evaluate the recycling value of the material categories in MSW. The recycling value of the MSW is determined using Equations (3) and (4), which denoted the recycling market price of the material as a basis. The prevailing recycling market price, the cost of each material is calculated using the prevailing data.

Recycling market price, allowing a complete assessment of the recycling market value of the waste material. There are different recycling market values if the end goal is upcycling rather than recycling. The recycling value however the amount demonstrates the price in Table 3. A basic market survey was carried out to identify recycling material value. Information was collected via a questionnaire survey to the waste collection partners. Furthermore, the recycling market value at the dumpsite is different from the value at the town level. The average recycling market value is taken in the protocol. User of the method can decide the source of recycling market value.

**Table 3:** Average weight (kg) of waste categories.

Composition	Waste Category	Mean composition weight kg	Composition%
W1	Paper and cardboard	13.42	27.53%
W2	Glass	0.28	0.58%
W3	Clothes	4.21	8.65%
W4	Clothes-leather	0.36	0.73%
W5	WEEE	0.55	1.12%
W6	Fuel (coconut husk)	2.03	4.17%
W7	Plastics-Mixed	13.43	27.55%
W8	HDPE (high-density polyethylene)	2.58	5.29%
W9	PP (polypropylene)	1.56	3.21%
W10	PS (polystyrene)	1.79	3.67%
W11	Rubber waste	0.62	1.26%
W12	Metals	1.49	3.06%
W13	Other non-biodegradable-offensive waste	0.55	1.12%
W14	Mixed municipal waste-Hazardous	0.39	0.80%
W15	Mixed municipal waste-General	5.49	11.27%

Wi = weight of recyclable material type i available in the 1 m^3^ vol

Ci = Unit cost(1 kg) of recyclable material i

Ce =Total recyclable value of waste in 1m^3^ vol

(3) Theeconomicvalueofthevolumeof1m3(Ce)=amountofrecyclablematerial(Wi)×materialpurchasingcostbycollectors(Ci)

(4) $$Ce\;=\sum_{i=1}^{i}Wi\;Ci\;$$

## Protocol validation

**Fig. 3:**
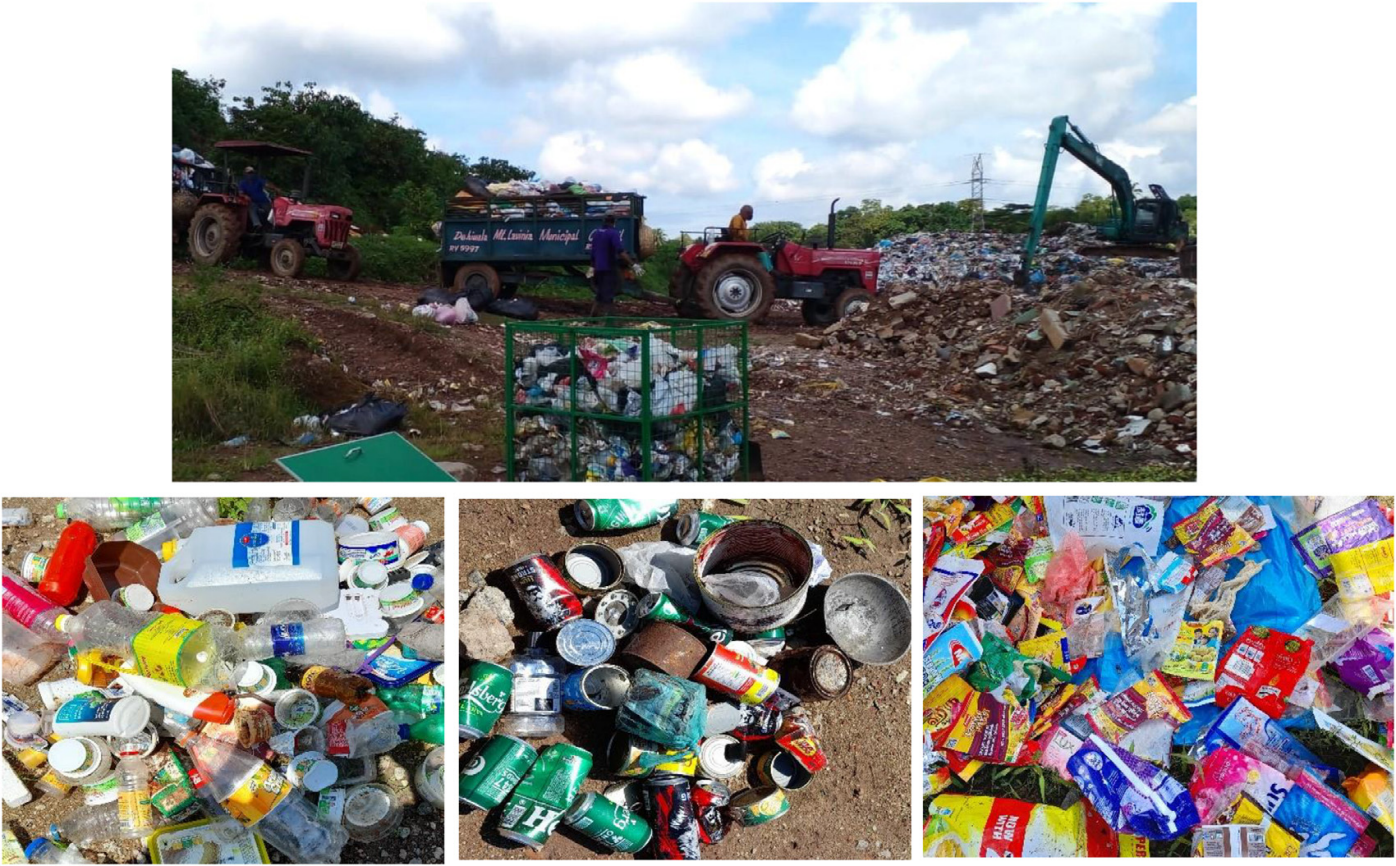
Mixed MSW at the dump site at Karadiyana, filled to one cubic meter frame and waste categories.

The investigation under study was conducted at the Karadiyana waste collection site. It is the largest MSW collection site in Sri Lanka, located in the southern part of Colombo city, which is the commercial capital of Sri Lanka. The MSW of eight local authorities was managed through this site with a mixed waste generation of 600 MTs daily. Mixed municipal solid waste dumps at a site daily (Fig. 3). The collection channel accepts only dry nonbiodegradable waste. The 250 MT truck fleet of 250 carry approximately 600 MTs of mixed waste daily to the current site for processing. At present, a portion of the waste is sorted by waste pickers and directed toward a recycling pathway, while another part is sent to the waste-to-energy plant after initial sorting. There isn't any mechanism for proper segregation and the evaluation of the recycling value of the waste materials.

Furthermore, 62% of the waste is biodegradable material [13], managed through the composting operation at the facility. Most waste materials are left in the landfill at the site. Nevertheless, the associated costs are imposing a substantial burden on waste management sites. Furthermore, the material recycling value of the contents in the waste category is not fully utilized, and the material ends up at the open dump.

### Composition of materials

The mean compositions of 60 samples were analyzed in weight to derive the material composition of the One cubic meter waste volume. The main composition categories at the Karadiyana waste site are mixed plastic 27.55%, paper and cardboard 27.53%, mixed waste 11.08% and clothing 7.73%. The main waste categories above 3 kg in weight in one cubic meter volume represent 70% of the composition, shown in Table 3, the waste categories are represented in W1 to W15.

### Calculation of the recycling value

As per this study, the largest contributors to landfill waste categories are plastic, paper, and cardboard waste streams. The plastic waste stream was the largest contributor to landfills, flowing by paper and cardboard. Hence, recyclers and potential businesses entering the resource recovery would be more tending to invest in plastic waste recovery rather than other waste categories, waste that has a much lower recycling value per unit weight of the waste being processed.

**Fig. 4:**
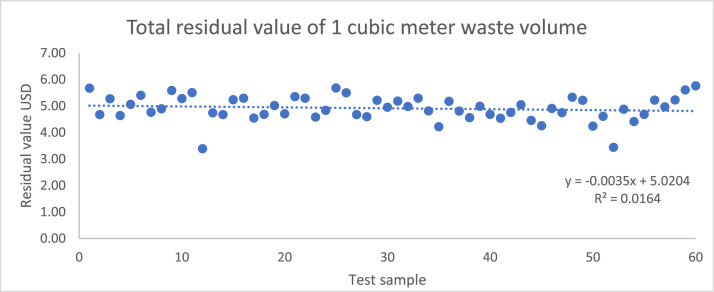
Total recyclable value of 1 m^3^ of waste materials as of Aug 2023 for 60 test samples.

The recycling value of the Wi weight of the waste material was obtained from the value of the recyclable market value of the period. (C1 to Cn are recyclable values of the respective components W1 to Wn shown in Tables 3 and 4) . The total recyclable value of one cubic meter sample has arrived at *Ce,* as mentioned in Eqs. (3) and (4). Fig. 4 represents the total C (C _total_) of the samples tested. It varies between USD 3.39 and USD 5.76 per cubic meter of waste volume.

**Table 4:** Recycling market value of waste categories as of 2023 August.

Economic value	Waste Category	Mean Value USD/Kg
C1	Paper and cardboard	0.08
C2	Glass	0.17
C3	Clothes	0.12
C4	Clothes-leather	0.07
C5	WEEE	0.40
C6	Fuel (coconut husk)	0.02
C7	Plastics-Mixed	0.28
C8	HDPE (high-density polyethylene)	0.28
C9	PP (polypropylene)	0.28
C10	PS (polystyrene)	0.28
C11	Rubber wastage	0.03
C12	Metals	0.12
C13	Other non-biodegradable-offensive wastages	0.00
C14	Mixed municipal waste-General	0.00
C15	Mixed municipal waste-Hazardous	0.00

### Recycling value change along the waste material supply channel

**Fig. 5:**
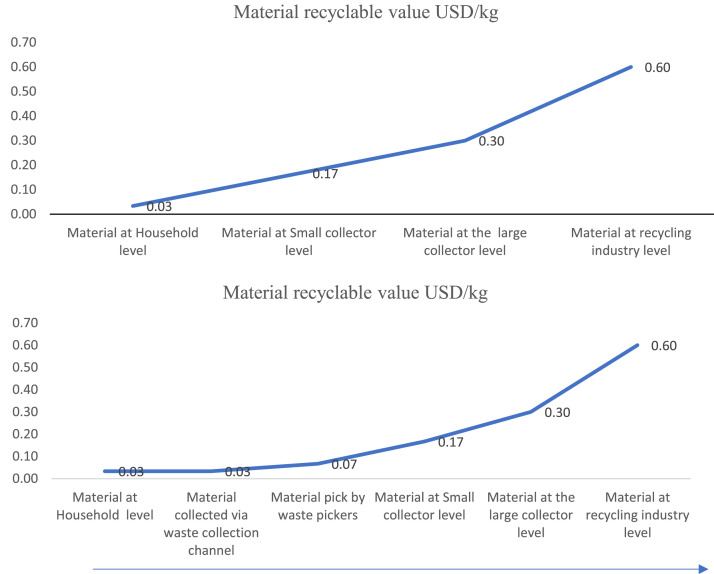
a & b- Material recyclable value variation along the supply channel (Value at the gate-in point).

It is imperative to distinguish fluctuations in the value of waste materials across diverse points within the material supply chain. Take plastic waste, for instance: originating at the household level, it undergoes subsequent transitions to either small-scale collectors or urban waste collection channels. In the scenario of routing to a small collector, the material progresses to larger collectors and ultimately arrives at plastic recycling facilities as per Fig. 5a. Conversely, if it follows an urban waste collection trajectory, it culminates in waste dumpsites such as the Karadiyana waste management facility, where waste pickers engage in collection. Subsequently, the plastic waste is traded either to minor waste collectors or major-scale counterparts before ultimately reaching a recycling facility (Fig. 5b). Fig. 5a and b explain a representative study example concerning plastic waste. Noteworthy from the study is the revelation that the most substantial value augmentation occurs through the involvement of large-scale collectors and recyclers.

### Sensitivity and uncertainty of the results

Sensitivity analysis (SA) has historically been an essential but informal pillar supporting scientific discovery and human decision-making. Its origins trace back to the invention of the law of friction by Leonardo da Vinci in the 15th century, as noted by [14].

Moreover, sensitivity analysis offers insights into the model's behavior under diverse conditions, uncovering any nonlinear or non-intuitive relationships between inputs and outputs. Decision-makers can utilize sensitivity analysis to prioritize data collection efforts or refine the model based on variables that exert the greatest influence on the outcomes. In the study, sensitivity analysis was conducted under two scenarios: (1) +/- 10% of volume, where sensitivity stood at 1, and +/- 10% of recycling material value, with sensitivity at 0.005. Results indicated that volume exhibited the highest sensitivity among the considered variables, whereas Recycling material revenue displayed the lowest sensitivity. These results are based on the specified scenario.

Uncertainty analysis can be conducted using techniques such as Monte Carlo based simulation techniques [15]. Results of the proposed method would further improve applying the uncertainty techniques based on the application.

### Model applicability

The protocol aims to standardize a methodology for comprehensively characterizing the composition, quantities, and potential recycling value of waste generated in a predefined geographical area. This comprehensive analysis serves as a foundational element for devising business models focused on transforming waste into valuable resources, fostering material symbiosis within the circular economic paradigm. The method's application offers a basis for simulating the hidden recycling value embedded within MSW samples, providing invaluable support to strategy developers, policymakers, and entrepreneurial ventures.

Moreover, evaluating the composition of a substantial waste dumpsite on a weight basis is deemed impractical. The proposed methodology, employing the 1m^3^ volumetric assessment approach, exhibits flexibility in characterizing the composition at a smaller scale and subsequently extrapolating the findings to encompass the volumetric intricacies of a larger dumpsite.

Additionally, the study recognizes that waste material value differs significantly at various points along the material supply chain. Consequently, the recycling value exhibits variations throughout the waste supply channel, from households to the final waste management destination.

However, it's crucial to note that the protocol does not encompass the density variations and material decay factors of MSW in waste dumpsites, which could be potential areas for further investigation to enhance the methodology's comprehensiveness and applicability

## Conclusion

The investigation introduces a novel testing methodology explicitly crafted for the evaluation of waste composition within a prescribed volume of one cubic meter. Its distinctiveness lies in the utilization of a fixed volume (1m^3^) to estimate the weight of waste materials, particularly applicable in scenarios where direct weight measurements pose challenges. This method facilitates the computation of recycling value, thereby playing a pivotal role in the formulation of business models harmonized with a circular economic framework.

A fundamental facet of the envisaged methodology is its inherent adaptability across diverse junctures within the waste collection continuum, spanning from initial household waste collection to the ultimate phases of waste management. This flexibility empowers the `one cubic meter' method to discern waste composition at any point along the waste supply chain, thereby fostering a nuanced comprehension of the dynamic evolution in composition and the associated potential recycling value. Understanding these fluctuations provides critical insights into optimizing resource recovery and valorizing waste materials across the entire waste management spectrum.

This, in turn, offers invaluable support to strategy developers, policymakers, and entrepreneurial ventures engaged in the sustainable management of household solid waste. In essence, this study establishes the groundwork for a comprehensive understanding of MSW composition and its recyclability, facilitating informed decision-making in the pursuit of a circular economy.

## CRediT authorship contribution statement

**N. Arosha Hemali:** Conceptualization, Methodology, Writing – original draft. **A.A.P. De Alwis:** Supervision, Validation, Writing – review & editing. **M. Wijesundara:** Visualization, Investigation. **L.G.L.M. Edirisinghe:** Formal analysis, Data curation.

## Declaration of competing interest

The authors declare that they have no known competing financial interests or personal relationships that could have appeared to influence the work reported in this paper.

## Data Availability

Data will be made available on request.
